# Enhanced secretion of a methyl parathion hydrolase in *Pichia pastoris* using a combinational strategy

**DOI:** 10.1186/s12934-015-0315-4

**Published:** 2015-08-28

**Authors:** Ping Wang, Lu Huang, Hu Jiang, Jian Tian, Xiaoyu Chu, Ningfeng Wu

**Affiliations:** Biotechnology Research Institute, Chinese Academy of Agricultural Sciences, Beijing, 100081 People’s Republic of China

**Keywords:** Methyl parathion hydrolase, Cytochromes heme binding domain, KKXX retrieval signal, Acid stability, Secretory expression, *Pichia pastoris*

## Abstract

**Background:**

Although *Pichia pastoris* has been successfully used to produce various recombinant heterologous proteins, the efficiency varies. In this study, we used methyl parathion hydrolase (MPH) from *Ochrobactrum* sp. M231 as an example to study the effect of protein amino acid sequence on secretion from *P. pastoris*.

**Results:**

The results indicated that the protein N-terminal sequence, the endoplasmic reticulum (ER) retention signal (KKXX) at the protein C-terminus, and the acidic stability of the protein could affect its secretion from *P. pastoris*. Mutations designed based on these sequence features markedly improved secretion from *P. pastoris*. In addition, we found that the secretion properties of a protein can be cumulative when all of the above strategies are combined. The final mutant (CHBD-DQR) designed by combining all of the strategies greatly improved secretion and the secreted MPH activity of CHBD-DQR was enhanced up to 195-fold compared with wild-type MPH without loss of catalytic efficiency.

**Conclusions:**

These results demonstrate that the secretion of heterologous proteins from *P. pastoris* could be improved by combining changes in multiple protein sequence features.

**Electronic supplementary material:**

The online version of this article (doi:10.1186/s12934-015-0315-4) contains supplementary material, which is available to authorized users.

## Background

The methylotrophic *Pichia pastoris* has been used extensively and successfully for the secretion of expressed recombinant proteins, because of its high expression level, stability, heredity, and mature fermentation process [1, 2]. The high-density fermentation and rapid growth of yeasts have had a great impact on the large-scale industrial production of foreign proteins, in which secretory expression is critical for simplifying the downstream protein purification process [3]. Therefore, the intrinsic commercial value of heterologous proteins has driven a wide range of studies on optimizing yeast secretion systems as “cell factories” [4]. Most studies on yeast secretion systems focused on the vector systems [5], the host strain [6], or its cultivation conditions [7], especially promoters [8, 9], signal peptides [10, 11], codon usage [12, 13], gene copy number [14], proteases [15], and chaperones [16]. Although extensive trials have been conducted, in some cases, secretion of the product into the culture supernatant remains low for some proteins [1, 4]. Strain engineering by genetic modification has become the most useful and effective method to overcome the drawbacks of yeast secretion pathways [17]. Although these methods can effectively enhance the expression of some proteins, the efficiency is variable and it is difficult for some foreign proteins to achieve optimal secretory expression in *P. pastoris*. Therefore, we hypothesized that some factors affecting secretion exist in the internal regions of proteins [18].

In this study, we used a methyl parathion hydrolase (MPH) from *Ochrobactrum* sp. M231 as an example to study the effect of the amino acid sequence of a protein on its secretion from *P. pastoris.* The MPH gene from *Ochrobactrum* sp. M231, isolated in our laboratory [19], can efficiently and specifically degrade methyl parathion, but it is little secreted from *P. pastoris* when expressed heterologously. However, another organophosphorus hydrolase, OPHC2 from *Pseudomonas pseudoalcaligenes*, also isolated in our laboratory [20], has a similar three-dimensional structure to MPH and it was over-expressed and efficiently secreted from *P. pastoris* [20, 21]. In addition, the *Pichia pastoris* genome does not contain a gene homologous to *mph* and the enzymatic activity of MPH was easy to measure. Therefore, MPH was used as a model to investigate the effect of internal protein factors on secretion when expressed in *Pichia pastoris*.

We focused on the effect of three internal protein factors on secretion. The first factor is the protein N-terminal sequence. The protein N-terminal sequence is important for its secretion [18, 22]. In this study, we utilized a strategy to fuse a small protein partner at the N-terminus. We combined this fusing strategy with other factors to assist secretion, which is different from the techniques used in the previous study [18]. We employed three fusion partners that were previously selected as secretion enhancers in bacteria and yeast: the maltose-binding protein (MBP) [23], glutathione-*S*-transferase (GST) from *Schistosoma japonicum* [23], and the cellulose-binding domain from *Trichoderma reesei* (CBD) [24]. In addition, we also evaluated the effect of a small protein tag, about 100 N-terminal amino acids of the cytochrome heme-binding domain (CHBD) [25], on enhancing the secretion of MPH. The second factor is the endoplasmic reticulum (ER) retention signal (HDEL or KKXX) located at the protein carboxy terminal (C-terminal). HDEL or KKXX is a short C-terminal signal that plays a crucial role in the localization of many soluble proteins in the endoplasmic reticulum of eukaryotic cells. Many ER proteins maintain their residence by dynamic retrieval from downstream compartments of the secretory pathway [26]. Sequence analysis of MPH revealed that it has a KKXX signal at its C-terminus, while OPHC2 does not. Therefore, we designed three mutants to knock out the signal and tested its effect on protein secretion. The third factor is the acidic stability of the protein. The pH of the fermentation medium of *Pichia pastoris* is usually below 5.5, and this may affect the accumulation of secreted proteins in the culture supernatant if they are not stable at low pH. We constructed the MPH mutant K277D, which has improved acidic stability in a previous study [27]. This mutant was used to evaluate its contribution to protein secretion in a low pH environment.

The aims of this study were to examine the effect of the above-mentioned factors on the secretion efficiency of MPH and evaluate the cumulative effects of each factor on secretion.

## Results

### Evaluation of single factors on the secretion in *P. pastoris*

To investigate the effects of protein N-terminal sequence, ER retention signal (KKXX) at the protein C-terminus, and acidic stability of the protein on the secretory expression of MPH from *P. pastoris*, pPIC9-based yeast expression constructs were generated. One hundred His^+^ transformants from the wild type and each mutant were analyzed for MPH production and the secretion using the standard enzyme assay.

**Fig. 1:**
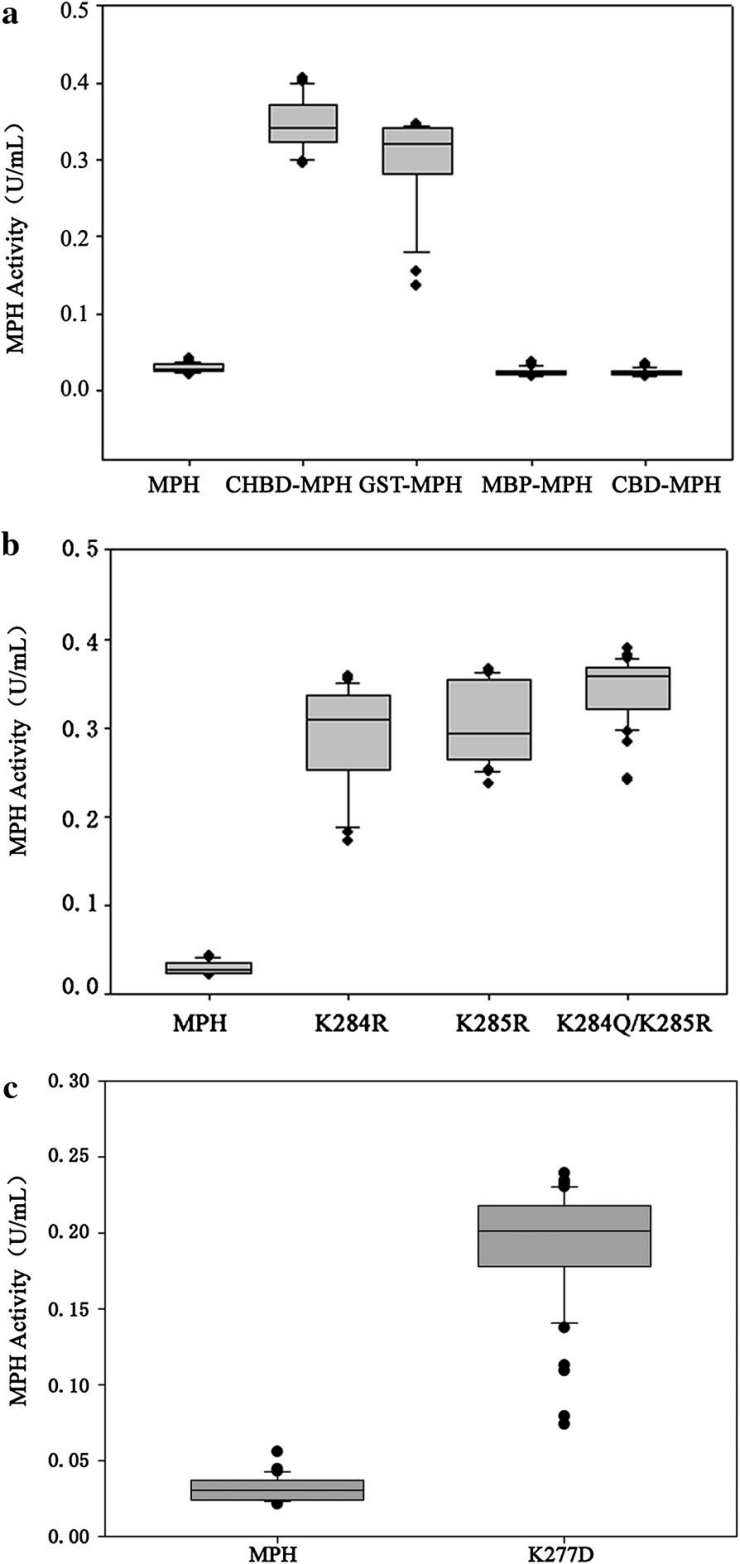
*Box*–*whisker plot* of MPH activity of single factors mutants in culture supernatants of *P. pastoris*. MPH activity of transformants in culture supernatants was determined using standard enzyme assays after 48 h of induction by methanol. **a** wild-type MPH and mutants of N-terminal tag-fusion MPHs: CHBD-MPH, GST-MPH, MBP-MPH, and CBD-MPH; **b** wild-type MPH and KKXX motif mutants: K284R, K285R, and K284R/285R; **c** wild-type MPH and acidic stability enhanced mutant: K277D. *Boxes* denote the interquartile range (IQR) between the first and third quartiles, while the *line within the box* denotes the median. *Whiskers* denote the lowest and highest values within 1.5-fold of the IQR from the first and third quartiles while circles denote outliers

The four tag-fusion MPHs, named CHBD-MPH, GST-MPH, MBP-MPH, and CBD-MPH, were constructed to assess the effects of protein N-terminal sequence on the secretory expression of MPH in *P. pastoris*. To facilitate the in vivo processing of the fusion proteins, a *kex2* endopeptidase-cleavage site was introduced between the fusion partners and MPH, and then all tags were excised in *P. pastoris* during secretion. During the evaluation of the effect of each factor on secretion from *P. pastoris*, we used a statistical criterion, the median of the box-whisker plot of MPH activity, to represent the secretory expression capacity of the mutants. As shown in Fig. 1a, the median MPH activities of the supernatants from the CHBD-MPH and GST-MPH transformants were 0.34 and 0.32 U/mL, respectively. In contrast, median MPH activity of the supernatants from wild type MPH transformants was 0.027 U/mL. Fusion of CHBD resulted in a maximal increase of extracellular MPH compared to the wild type. Also, the GST tag improved the secretion of MPH protein from *P. pastoris*. In contrast, no effect was observed with the MBP and CBD fusion partners. SDS-PAGE confirmed these results (data not shown). These results indicated that N-terminal protein tags could significantly enhance the secretion of MPH from *P. pastoris*, and CHBD was used as tag in the following experiments.

Three mutants (K284R, K285R, and K284Q/K285R) were constructed to disrupt the ER retention signal (KKXX) located at the C-terminus of MPH and evaluate its effect on the secretory expression of MPH from *P. pastoris*. To minimize other unnecessary effects, lysine (K) was mutated to the similar amino acid arginine (R), producing the two mutants K284R and K285R. Meanwhile, due to the similar protein construction and function, but distinct secretion from *P. pastoris* of MPH and OPHC2, we also mutated the KK in MPH to the corresponding amino acids QR in OPHC2, in addition to disrupting the KKXX signal. As shown in Fig. 1b, the median MPH activity of the supernatants from the three mutant transformants was higher than 0.3 U/mL. In contrast, the supernatant MPH activity of wild type MPH transformants was ~0.03 U/mL. These results indicated that disruption of the KKXX signal could markedly increase the secretion of MPH from *P. pastoris*, which was confirmed by SDS-PAGE (data not shown). Based on the enzyme activity (Fig. 1b) of the WT and mutants, K284Q/K285R was used for the next experiment.

The K277D mutant was constructed to evaluate the acidic stability of protein to the secretory expression of MPH from *P. pastoris*. As shown in Fig. 1c, supernatant from K277D transformants had a median MPH activity of up to 0.2 U/mL. In contrast, supernatant from wild-type MPH transformants had activity of ~0.03 U/mL. These results indicated that improving the acidic stability of MPH could improve its secretion from *P. pastoris*, which was confirmed by SDS-PAGE (data not shown).

### Effect of the combination of factors on secretion from *P. pastoris*

**Fig. 2:**
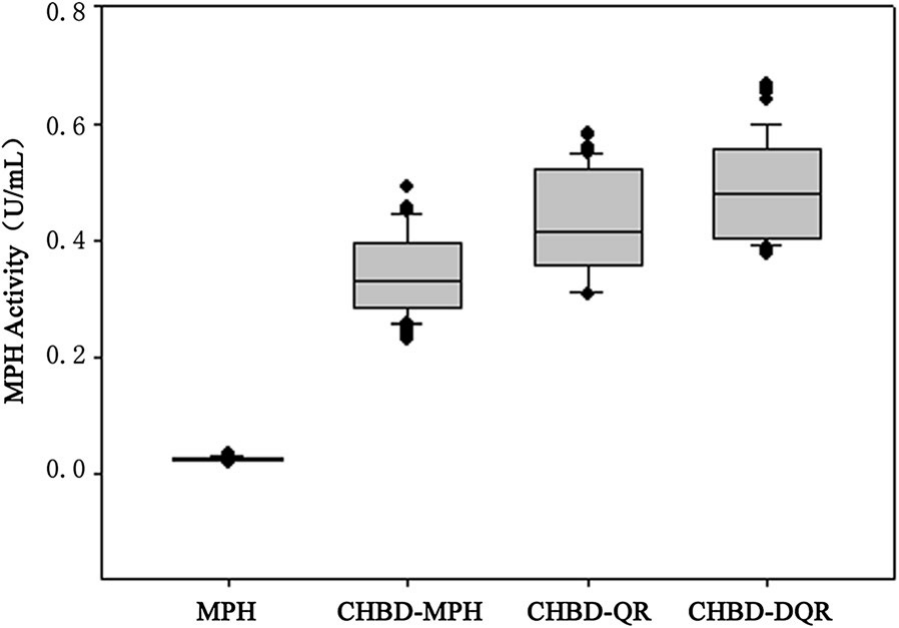
*Box*–*whisker plot* of MPH activity of combined factors in culture supernatants of *P. pastoris*. *Boxes* denote the interquartile ranges (IQR) between the first and third quartiles, while the *lines within the boxes* denote the medians. *Whiskers* denote the lowest and highest values within 1.5-fold of the IQR from the first and third quartiles, while *circles* denote outliers

After all factors were examined, we sequentially combined them to evaluate the cumulative effect of the individual factors on the secretion of MPH from *P. pastoris*. We chose the median of the box-whisker plot of MPH activity to represent MPH activity and the secretion capacity of mutants. As shown in Fig. 2, fusion with CHBD (CHBD-MPH) resulted in a significant increase in extracellular MPH activity to 0.33 U/mL. The combination of the KKXX signal mutant with CHBD-MPH (CHBD-QR) resulted in a 1.2-fold increase in secretion to 0.41 U/mL compared to CHBD-MPH. Finally, CHBD-DQR, the combination of acid stability with CHBD-QR, resulted in further enhanced accumulation of activity in the extracellular medium, to 0.48 U/mL. All data were analyzed statistically using t tests, which confirmed the results (p = 0.00). The results indicate that each of the individual factors has a cumulative effect on the secretion of MPH from *P. pastoris.*

### Production and activity of wild type and mutant MPH proteins

**Fig. 3:**
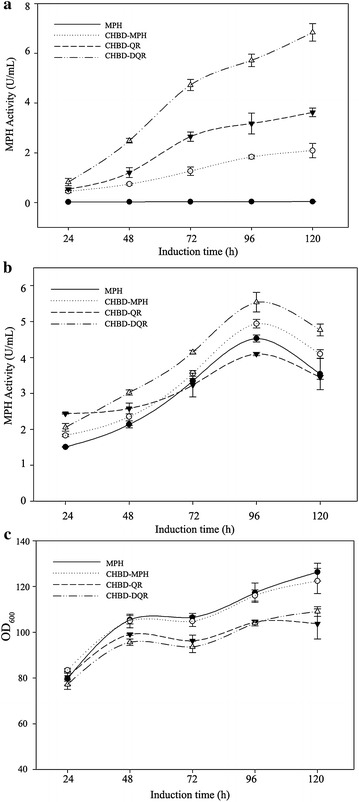
Enzyme activity and growth kenetics of the recombinant *P. pastoris* clones in shake-flask culture. Transformants expressing MPH (solid circle), CHBD-MPH (*hollow circle*), CHBD-QR (*solid triangle*), or CHBD-DQR (*hollow triangle*) were induced by methanol for the indicated times on the x-axis, and MPH activity was determined using standard enzyme assays (as indicated as y-axis). **a** Extracellular MPH activity of the recombinant *P. pastoris* clones; **b** intracellular MPH activity of the recombinant *P. pastoris* clones; **c** Growth kenetics of the recombinant *P. pastoris* clones. Enzyme activity is expressed as the mean of three samples, and error bars indicate standard deviations (SD)

**Fig. 4:**
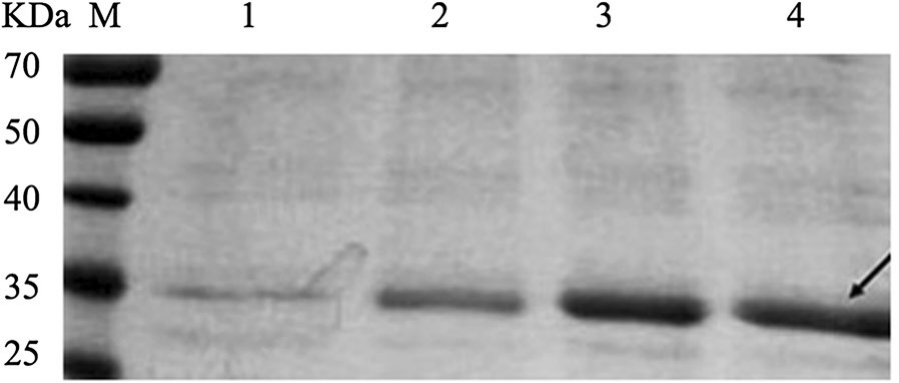
SDS-PAGE analysis of culture supernatants after 120 h. Transformants were induced by methanol as indicated, and the supernatants (pH = 6.0) after 120 h were analyzed by SDS-PAGE. Lane M, molecular weight marker; *lane 1* MPH; *lane 2* CHBD-MPH; *lane 3* CHBD-QR; *lane 4* CHBD-DQR. The positions of expressed MPH proteins are indicated by *arrows*

The selected four transformants (MPH, CHBD-MPH, CHBD-QR, CHBD-DQR) were cultured in a shake flask to measure MPH expression and secretion. Maximum secretion yield was detected after 5 days of methanol induction at 28 °C. The CHBD-MPH culture exhibited activity of 1.82 U/mL, approximately 50-fold that of MPH (Fig. 3a). The MPH variants CHBD-QR and CHBD-DQR exhibited significantly increased activities of 3.62 and 6.84 U/mL (103- and 195-fold increases), respectively, compared with wild-type MPH. In contrast, the variants exhibited an intracellular activity similar to the wild type (Fig. 3b). The different secretion, similar intracellular activity (Fig. 3b) and distinct extracellular activity (Fig. 3a), revealed that all those factors improved the secretion of MPH from *P. pastoris.* After 120-h induction with methanol, the supernatant proteins were subjected to SDS-PAGE analysis (Fig. 4). The CHBD-DQR mutant showed prominent protein binding at ~35 kD, and the intensity of protein bands in SDS-PAGE gels were correlated with the MPH enzyme activity in the culture supernatant.

During the 5-day induction period, the MPH and CHBD-MPH transformants had similar growth rates while the CHBD-QR and CHBD-DQR transformants exhibited lower growth rates after 24 h of induction (Fig. 3c). And the wild type MPH stain exhibited 21 %, 15 % higher than CHBD-QR and CHBD-DQR stains in final OD levels (Additional file 1: Table S1). These data suggested that the increased MPH levels produced by the three mutants were not due to higher cell densities or enhanced proliferation. Moreover, if divided by the cell densities (OD_600_), the variants CHBD-QR and CHBD-DQR exhibited more increased activities (125- and 225-fold increases) compared with wild type MPH strain (Additional file 1: Table S1).

### Determination of gene copy number and MPH mRNA levels

We analyzed the relative MPH transcriptional levels of the representative MPH, CHBD-MPH, CHBD-QR, and CHBD-DQR transformants to determine whether differences in transcription contributed to the differential protein expression. After induction with methanol for 24 h, quantitative real-time PCR revealed that mRNA expression of CHBD-DQR was lowest among the four representative transformants. CHBD-MPH, CHBD-QR, and CHBD-DQR were 143, 124, and 56 %, respectively, of the MPH mRNA level (Table 1). These relatively sm all changes in mRNA expression clearly would not lead to the differences in protein secretion, particularly the decreased mRNA level would not contribute to the highest extracellular MPH activity of CHBD-DQR. Gene copy numbers can affect gene expression and secretion in *Pichia pastoris,* so copy numbers of the *mph* gene in WT and mutant transformants were determined by quantitative PCR; the selected four transformants each contained a single copy of *mph* (Table 1). Overall, the differences in protein secretion were not an effect of gene copy number or MPH mRNA levels. Thus, the differences in protein secretion were likely regulated at the posttranscriptional level.

**Table 1:** Investigation of the relationships among MPH secretion, gene dosage, and relative mRNA expression

Strain name	Gene copy number	Relative mRNA expression^a^
MPH	1	1
CHBD-MPH	1	1.43
CHBD-QR	1	1.24
CHBD-DQR	1	0.56

^a^MPH/GAPDH ratio

### Kinetic characterization and thermostability of wild-type and mutant enzymes

The kinetics and thermostability of the MPH activities were determined using a protocol described previously [28]. The results are shown in Table 2. All of the mutants had catalytic efficiencies similar to the wild-type enzyme. Thus, the distinct extracellular activities between wild type and mutants (Fig. 3a) were not resulted from the slight differences in catalytic efficiencies (Table 2).

**Table 2:** Kinetic parameters of WT and mutant MPH

Enzyme	*k*_cat_ (min^−1^)	*K*_m_ (μM)	*k*_cat_/*K*_m_ (μM^−1^ min^−1^)
CHBD-MPH	215.20 ± 7.30	86.85 ± 5.85	2.48
CHBD-QR	329.50 ± 12.92	119.10 ± 8.26	2.77
CHBD-DQR	572.80 ± 12.30	168.50 ± 7.57	3.40

## Discussion

*Pichia pastoris* is an important and useful expression system for the secretion of target proteins and can efficiently produce heterologous proteins at low cost. However, some proteins cannot be secreted from *P. pastoris*, MPH from *Ochrobactrum* sp. M231 is one of the example that had limited secretion when tentatively expressed in *P. pastoris* [18, 29], while another organophosphorus hydrolase, OPHC2, a similar three-dimensional structure to MPH, is secreted efficiently [20]. In this study, we used MPH as a model to engineer amino acid sequences to improve the secretion of foreign proteins from *P. pastoris.* The results suggest that there are some protein features that may affect secretion. The usual strategies [6–8, 30–33] did not resolve the secretion problems of MPH and few studies related to protein secretion from *P. pastoris* had focused on the amino acid sequences of proteins [12, 34]. With luck, the OPHC2 protein provided useful information for our study. The approaches about improving MPH secretion in this study could be applied to other foreign proteins that have secretion obstacle in *P. pastoris*.

In a previous study, we found that the N-terminus is related to the secretion of MPH from *P. pastoris*. When the N-termini of OPHC2 and MPH were swapped, the secretion of MPH from *P. pastoris* was improved, but catalytic efficiency was lost [18]. Here, we added four common fusion tags (CHBD, GST, MBP, CBD) in front of the N-terminal sequence of MPH, and designed a *kex2* endopeptidase-cleavage site between the fusion partners and MPH to prevent possible loss of protein catalytic efficiency. Fusion of CHBD or GST resulted in a marked increase in extracellular MPH. This had been explained by the ability of tags to act as a chaperone within the context of a fusion protein, and promote proper folding of the fusion partner [23]. However, unlike previous studies [23, 35], fusion of MBP or CBD did not enhance the secretion of the recombinant protein. The results in this study suggest that this strategy improves MPH secretion and that selection of tags is critical, because different tags appear to target different proteins.

A foreign protein that crosses the ER membrane must be exported to the Golgi apparatus. As described above, ER efficient export could be achieved by altering the ER retention signal (KKXX) located at the protein C-terminus [26, 36]. A KKXX signal sequence was found at the MPH C-terminus, but not that of OPHC2. Disruption of the KKXX signal could decrease the retention of MPH, thus significantly improved the secretion of MPH (Fig. 1b). This strategy is important for expression of prokaryotic, synthetic or ER-retained proteins in eukaryotic strains.

Another factor that might account for low secretion level could be degradation of the secreted protein under low pH environment, especially during a long fermentation period. The pH of the fermentation medium of *P. pastoris* is usually below 5.5, while the instability property of wild-type MPH at this low pH might result in low secretion in *P. pastoris*. We hypothesized that improving the acidic stability of MPH would enhance its secretion, which was confirmed by the results from this (Fig. 1c). We believe this strategy has broad application for improving the secretion of foreign proteins in *P. pastoris* although few efficient methods to improve the protein acidic stability are available [27, 37, 38].

Besides, as shown in Fig. 2 and Fig. 3a, these results revealed that each of the individual factors has a cumulative effect on the secretion of MPH from *P. pastoris*, which was also confirmed by SDS-PAGE (Fig. 4). The date of the growth rates, mRNA expression, gene copy number and MPH mRNA levels of the wild-type and mutants indicated that these did not cause differences in protein secretion. Additionally, enzyme properties of the wild type and mutants were analyzed. They had similar catalytic efficiencies (Table 2), thermostability, optimal pH and temperatures compared to wild type (data not shown), which indicated that enhanced secretion of the mutant proteins did not result from protein thermostability [39] or higher catalytic efficiencies. In conclusion, all of these results indicated that these factors markedly enhanced MPH secretion from *P. pastoris.*

Therefore, a similar approach could be used to enhance secretion of other proteins from *P. pastoris* using this two-step mutation strategy. The first step is to identify the individual sequence factors that affect secretion; the second step is to combine those factors. Although this study used only one protein as an example to identify sequence factors related to secretion, it could give implication for solving similar problems in other proteins.

## Methods

### Strains, plasmids, and reagents

The GenBank accession number of *Ochr*-MPH is ACC63894. The *P. pastoris* strain GS115 and expression vector pPIC9 were purchased from Invitrogen (Carlsbad, CA). The *E. coli* strain Top10 (TIANGEN Biotech, Beijing, China) was used for recombinant plasmid amplification and cells were cultured aerobically at 37 °C in Luria–Bertani medium containing 100 μg/mL ampicillin. Minimal dextrose medium (MD), buffered complex glycerol medium (BMGY), yeast extract peptone dextrose medium (YPD), and buffered complex menthol medium (BMMY) were prepared according to the manufacturer’s instructions (Invitrogen).

### Construction of mutants

The recombinant plasmid pPIC9-MPH constructed for expression was described previously [18]. To construct the recombinant pPIC9-CHBD-MPH plasmid, two DNA fragments were PCR amplified using the pPIC9-MPH plasmid as the template and two oligonucleotide pairs, pPIC9-F/MPH-R and MPH-F/pPIC9-R, as primers. Another DNA fragment encoding CHBD was amplified by PCR from the pET22b-CHBD plasmid using the primers CHBD-F and CHBD-R. To express the native mature *mph* gene without additional amino acids at the N-terminus, a linker sequence of the *kex2* signal cleavage site was introduced at the junction of CHBD and MPH. The PCR products were purified using a gel-extraction kit (TIANGEN Biotech, China). The resulting fragments were ligated by homologous recombination using the CloneEZ kit (GenScript, NJ, America). To disrupt the MPH KKXX signal, the amino acid K was replaced by the similar amino acid R for minimum interference. The mutants K284R and K285R were generated by PCR amplification using the primers K284R-F/K284R-R and K285R-F/K285R-R (Table 3), respectively. Meanwhile, the KK amino acids in MPH corresponding to QR in OPHC2 were mutated to generate the two point mutant pPIC9-CHBD-K284Q/K285R-MPH (CHBD-QR). To create CHBD-QR,two DNA fragments were amplified by PCR from pPIC9-CHBD-MPH using the two oligonucleotide pairs pPIC9-F/KK-R and KK -F/pPIC9-R and ligated using the CloneEZ kit. In the same manner, three other tag proteins (GST, MBP, and CBD) were fused to the N-terminus of MPH. The recombinant plasmid pPIC9-CHBD-K284Q/K285R-MPH (CHBD-DQR) was constructed using two oligonucleotide pairs, pPIC9-F/K277D-R and K277D -F/pPIC9-R, from CHBD-QR in a similar way. All primers used in this study are listed in Table 3. All mutated sites and ligation junctions in recombinant vectors were confirmed by DNA sequencing (State Key Laboratory for Crop Genetic Improvement, Chinese Academy of Agricultural Sciences, Beijing, China).

**Table 3:** Primer sequences used for the construction of mutants

**Primer name**	**Primer sequence** ^**a**^
pPIC9-F	5’-GTGCTCAACGGCCTCAACCTACTACTG-3’
pPIC9-R	5’-TAGGTTGAGGCCGTTGAGCACCGCCGC-3’
MPH-F	5’-GAAAAGCGTGAAGCCGAGGCCGCTGCTCCACAAGTTAGAACT-3’
MPH-R	5’-AGCTTCAGCCTCTCTTTTCTC-3’
CHBD-F	5’-*AAAAGAGAGGCTGAAGCT*ATGGCAGAACAAAGCGACAAG-3’
CHBD-R	5’-CTCGGCTTCACGCTTTTCAAGGGTTTCCGAAGGCTTGGC-3’
GST-F	5’-*AAAAGAGAGGCTGAAGCT*ATGTCCCCTATACTAGGTTAT-3’
GST-R	5’-CTCGGCTTCACGCTTTTCGTCACGATGCGGCCGCTCGAG-3’
MBP-F	5’-*AAAAGAGAGGCTGAAGCT*ATGAAAATAAAAACAGGTGCA-3’
MBP-R	5’-CTCGGCTTCACGCTTTTCTCCGCCAAAACAGCCAAGCTG-3’
CBD-F	5’-*AAAAGAGAGGCTGAAGCT*ATGAAAATCGAAGAAGGT-3’
CBD-R	5’-CTCGGCTTCACGCTTTTCTTGAAGCTGCCACAAGGC-3’
K284R-F	5’-GCTGCCGTTGAGAGA **CGT**AAAGCTTTCGCTGATGCC-3’
K284R-R	5’-TTT**ACG** TCTCTCAACGGCAGCAGACTTGGAGTCAGA-3’
K285R-F	5’-GCTGCCGTTGAGAGAAAG **CGT**GCTTTCGCTGATGCC-3’
K285R-R	5’-**ACG** CTTTCTCTCAACGGCAGCAGACTTGGAGTCAGA-3’
KK-F	5’-GCTGCCGTTGAGAGA **CAGCGT**GCTTTCGCTGATGCC-3’
KK-R	5’-**ACGCTG** TCTCTCAACGGCAGCAGACTT-3’
K277D-F	5’-TTGGACTCTGACTCC **GAT**TCTGCTGCCGTTGAG-3’
K277D-R	5’-**ATC** GGAGTCAGAGTCCAATTGGTT-3’

^a^The mutation sites are indicated by bold sequences. The 15-base sequences for homologous recombination are underlined. The DNA sequence constituting the *Kex2* signal cleavage site is italicized

### *pastoris* transformation and identification of transformants

*Bgl*II (Takara)-linearized recombinant vectors were transformed into *P. pastoris* GS115 by electroporation using the Gene Pulser system (Bio-Rad; conditions used: 2.5 kV, 25 μF, and 400 Ω). Transformants were initially grown on MD plates and then confirmed by colony PCR using the primers 5′AOX1 and 3′AOX1.

### Enzymatic properties of WT and mutant MPH

The purification and quantification of recombinant WT and mutant enzymes were performed as described previously [28]. The standard enzyme assay, and determination of the enzymatic properties and the kinetic stability of WT and mutant enzymes were performed as described previously [27]. The intracellular and extracellular activities of MPH from *P. pastoris* were measured as described previously [18].

### Selection of high producing recombinant *P. pastoris* strains

After transformation, the His^+^ transformants from the MD plates were grown in 3-mL BMGY and induced in 1-mL BMMY for 48 h. Then, 100 clones from each wild-type and mutant MPH transformant were assessed for the secretion of the expressed proteins using a standard enzyme assay. We also selected a high-producing recombinant *P. pastoris* strain for each gene from the shake-flask cultures.

### Expression of MPH and mutant proteins in shake-flask culture

The colonies of His^+^ transformants exhibiting MPH activity were inoculated into 45-mL BMGY at 28 °C, with constant shaking at 200 rpm, until the optical density at 600 nm (OD_600_) reached 5.0. Cell pellets were then resuspended in 15-mL BMMY and induced at 28 °C with constant shaking at 200 rpm for 120 h. Methanol was added to a final concentration of 0.5 % (v/v) every 24 h. The culture supernatant and cells were harvested by centrifugation (12,000*g*, 3 min, 4 °C) to analyze MPH activity, according to methods described previously [29]. MPH activity in the supernatant and cells was determined using a standard enzyme assay.

### Determination of gene copy number and transcriptional level using quantitative real-time PCR

The gene copy number was determined using qRT-PCR with the GAP gene as a reference. Genomic DNA was prepared using the TIANamp Yeast DNA kit (TIANGEN). Gene copy number was determined by quantitative PCR as described previously [40] using SYBR^®^ Green Real-time PCR Master Mix–Plus (Toyobo, Osaka, Japan). To construct the standard curves for *gapdh* and *mph*, fivefold serial dilutions of pGM-T-gapdh and pGM-T-mph ranging from 10^4^ to 10^8^ copies/μL were used and the Ct values were plotted against log values (copies of plasmid DNA). The concentration of the plasmid DNA was determined with a Nanodrop spectrophotometer. All real-time qPCR reactions were run in triplicate using the following program: 95 °C for 10 min, 45 cycles at 95 °C for 15 s and 60 °C for 30 s. Each 20-μL reaction contained 10-μL 2 × SYBR^®^ Green Real-time PCR Master Mix–Plus, 0.6 μL of 10 μM forward and reverse primers, 1.0 μL of genomic DNA and 7.8 μL of sterile deionized water. Absolute copy numbers for *gapdh* and *mph* were calculated using the mean Ct value and the corresponding gene standard curve.

RNA isolation was carried out with TRIzol (TIANGEN) following the procedures recommended by the manufacturer. Contaminating DNA was removed by digestion with DNase I (NEB) and verified by PCR. The absolute copy numbers of *mph* and *gapdh* mRNA were determined by qRT-PCR as described above.

## Additional file

Additional file 1:
**Table S1**. Comparison of the extracellular MPH activities of wild type and mutants in the supernatants after 120 h induction

## Abbreviations

BMGY: buffered complex glycerol medium
BMMY: buffered complex menthol medium
CBD: cellulose-binding domain
CHBD: cytochrome heme-binding domain
CHBD-QR: pPIC9-CHBD-K284Q/K285R-MPH
CHBD-DQR: pPIC9-CHBD-K277D-K284Q/K285R-MPH
ER: endoplasmic reticulum
MBP: maltose-binding protein
MD: minimal dextrose medium
MPH: methyl parathion hydrolase
K: lysine
R: arginine
X: a random amino acid
SD: standard deviation
YPD: yeast extract peptone dextrose medium
