# Supplementary material for: Enhanced secretion of a methyl parathion hydrolase in Pichia pastoris using a combinational strategy
Source: Microb Cell Fact. 2015 Aug 28;14:123. doi: 10.1186/s12934-015-0315-4 (PMC4551668; doi:10.1186/s12934-015-0315-4)
Supplement: Additional file 1: — Table S1. Comparison of the extracellular MPH activities of wild type and mutants in the supernatants after 120 h induction [file 12934_2015_315_MOESM1_ESM.docx]

**Table S1. Comparison of the extracellular MPH activities of wild type and mutants in the supernatants after 120 h induction**

|  | OD_600_ | Extracellular MPH activity（U/mL） | Fold^a^ |
| --- | --- | --- | --- |
| MPH | 126 ± 4 | 0.035 ± 0.002^b^ | 1 |
| CHBD-MPH | 122 ± 6 | 1.82 ± 0.28 | 54 |
| CHBD-QR | 104 ± 6 | 3.62 ± 0.17 | 125 |
| CHBD-DQR | 109 ± 2 | 6.84 ± 0.35 | 225 |

a：Due to the different growth rates of wild MPH and mutants, the ratio of extracellular MPH activity and OD_600_, named A is used to eliminate the effect of the cell density on extracellular activity. Therefore, the ratio, A of Mutants/ A of MPH, represented the real activity fold when compared with the wild MPH. A= Extracellular MPH activity / OD_600_; Fold= A of Mutants/ A of MPH.

b: Considering this value is very small and round the value will be a great influence on the result of Fold, we keep it to three decimal places.
